# Folate deficiency-triggered redox pathways confer drug resistance in hepatocellular carcinoma

**DOI:** 10.18632/oncotarget.4422

**Published:** 2015-06-27

**Authors:** Chun-Te Ho, Hung-Sheng Shang, Jin-Biou Chang, Jun-Jen Liu, Tsan-Zon Liu

**Affiliations:** ^1^ Graduate Institute of Medical Sciences, College of Medicine, Taipei Medical University, Taipei, Taiwan; ^2^ Department of Pathology, National Defense Medical Center, Division of Clinical Pathology, Tri-Service General Hospital, Taipei, Taiwan; ^3^ School of Medical Laboratory Science and Biotechnology, Taipei Medical University, Taipei, Taiwan; ^4^ Translational Research Laboratory, Cancer Center, Taipei Medical University and Hospital, Taipei, Taiwan

**Keywords:** chemotherapy, folate, GRP78, multi-drug resistance, hepatoma

## Abstract

Patients with hepatocellular carcinoma (HCC) are prone to folate deficiency (FD). Here we showed that, in cell line-specific manner, FD caused resistance to FD-induced oxidative stress and multi-drug resistance (MDR). This resistance was due to upregulation of glucose-regulated protein 78 (GRP78) and Survivin. Using siRNA and Epigallocatechin gallate (EGCG), we found that GRP78 and Survivin cooperatively conferred MDR by decreasing FD-induced ROS generation. Our data showed that FD increases GRP78 and Survivin, which serve as ROS inhibitors, causing MDR in HCC. We suggest that folate supplementation may enhance the efficacy of chemotherapy.

## INTRODUCTION

Folic acid (folate; vitamin B9) is an essential micronutrient and a critical coenzymes for the *de novo* synthesis of purine and thymidylate nucleotides and methylation and demethylation of homocysteine/methionine [1–3]. Folate receptor overexpressed in tumor tissues and disruption of one-carbon metabolism can cause oxidative stress-mediated DNA damage and apoptosis [4–9].

Hepatocellular carcinoma (HCC) is one of the leading cause of death [10], with low response to conventional chemotherapies [11, 12]. Most HCC patients are prone to folate deficiency [13–15]. Meenam et al. [16] observed that folate content was lower in tumors than the adjacent normal cells. Furthermore, dietary methyl deficiency and Hcy-aggravated hydrogen peroxide production have been demonstrated to be a pair of contributing factors in promoting mutagenesis and hepatocarcinogenesis [17, 18]. Importantly, Kuo et al. [15] reported that approximately 60% of HCC patients were deficient in folate and established that folate levels decreased drastically as HCC stage progressed. Thus, they suggested that low blood folate status could be a risk factor for tumor progression. These studies established that folate deprivation is a risk factor for HCC.

Nutritional deficiencies, such as folate deprivation, and chemotherapeutic drugs can cause cell death via generation of oxidative-nitrosative stress (ONS) [19–22]. However, some types of cancer cells exhibit increased reactive oxygen species (ROS) generation that may promote cell proliferation and in many cases can be coupled to redox adaptation (RA) to promote cell survival and drug resistance. RA can explain how cancer cells survive under persistent endogenous ROS stress and become resistant to certain anti-cancer agents. Thus far, research efforts focusing on whether or not folate deficiency (FD)-induced ONS on the induction of RA and its potential impacts on the MDR acquisition of HCC cells have been relatively sparse. Our data demonstrated that some poorly-differentiated and invasive subclone variant, such as SK-Hep-1 cells, could withstand FD-induced ONS via evading apoptosis and becoming MDR through RA-mediated upregulation of GRP78 and Survivin, which decrease oxidative stress and promote survival.

## RESULTS

### Folate deficiency (FD) can transform redox adaptation-prone hepatoma cell lines into MDR phenotype

**Figure 1:**
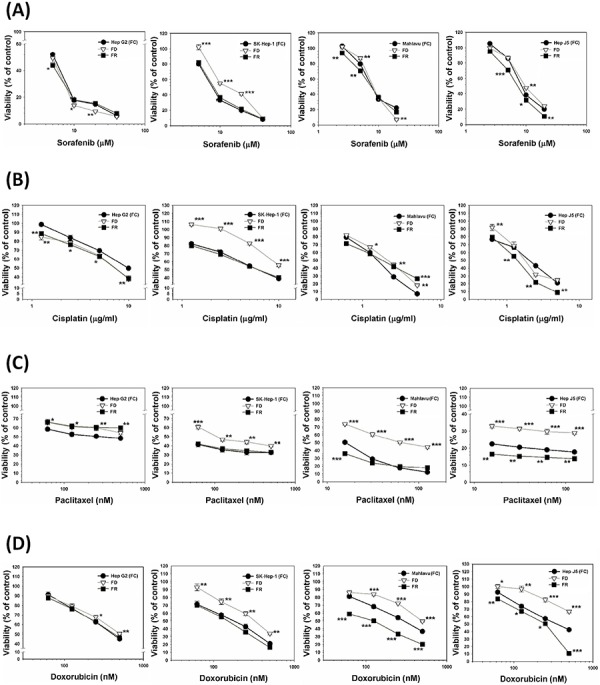
Comparison of relative sensitivities of a group of ROS-producing anti-cancer drugs against various HCC subclone variants cultivated in the presence or absence of folate and the effects of its resupplementation HCC subclone variants including Hep G2, SK-Hep-1, Mahlavu and Hep J5 cells were cultivated under either folate-sufficient (FC) or folate-deficient (FD) α-MEM media for one-week. After that, both FC and FD groups were treated with various concentrations of drugs including sorafenib **A.** cisplatin **B.** paclitaxel **C.** and doxorubicin **D.** Meanwhile, some FD cultured plates were replaced with FC medium and then similarly treated with all four drugs [designated as folate resupplemented group (FR)]. All the drug-treated cultured plates (FC, FD, and FR) were allowed to grow for additional 48-h and the viability of all cultured plates was then measured by SRB method. Remarkably, we observed that except for Hep G2 cells, all three other types of HCC cells cultivated under FD condition could transform themself into multi-drug resistant (MDR) phenotype. Even more interestingly, cells from FR group could apparently restitute themselves back to drug-sensitive attribute implying that folate micronutrient *per se* was capable of reversing MDR attribute. *p < 0.05; **p < 0.01; ***p < 0.001.

A group of HCC subclone variants including Hep G2, Hep J5, Mahlavu and SK-Hep-1 were cultivated under either FC or FD condition for one-week, followed by the treatment of these cells with various concentrations of a group of ROS-producing anti-cancer drugs including sorafenib, cisplatin, paclitaxel and doxorubicin. As judged by the viability data, except for Hep G2 cells (redox adaptation-null; Supplementary Figure 1), all three other HCC cell types cultivated under FD condition could unilaterally transform themselves into MDR phenotype. However, this acquired MDR attribute could apparently be restituted either partially or even completely by folate resupplementation (FR) (Figure 1). This finding implies that folate micronutrient *per se* can confer the cells with the capacity to reverse MDR acquisition. *p < 0.05; **p < 0.01; ***p < 0.001.

### FD-evoked MDR acquisition is linked to the redox adaptation (RA)-mediated upregulation of GRP78 and Survivin

**Figure 2:**
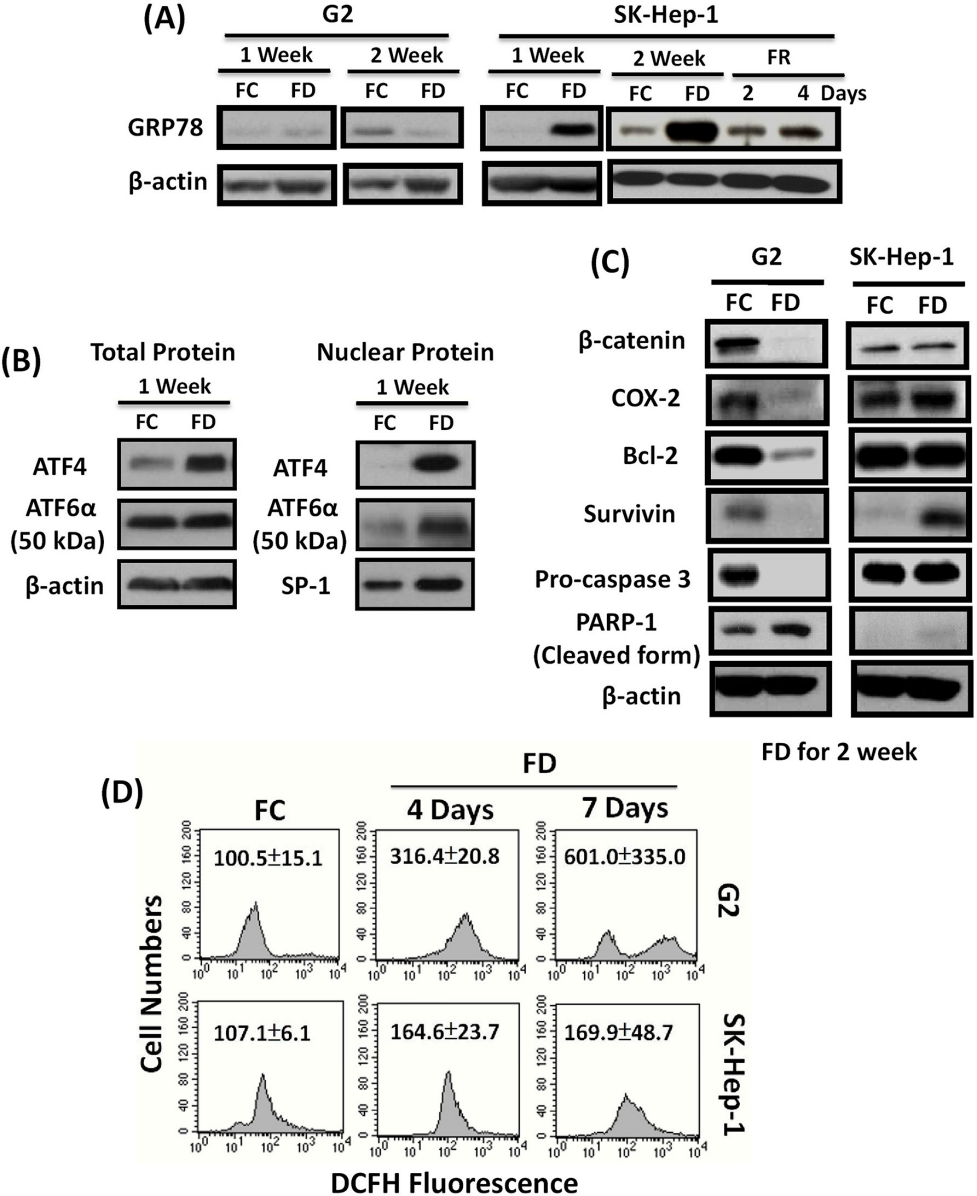
FD-induced multi-drug resistance (MDR) acquisition is mechanistically linked to redox adaptation (RA)-mediated upregulation of GRP78 and Survivin pathways SK-Hep-1 and Hep G2 cells were cultivated under FC or FD condition for two-week period. Using western blot technique, we identified that SK-Hep-1, but not Hep G2 grown under FD condition could prolifically overexpressed GRP78, an ER stress chaperone protein. Interestingly, folate resupplementation could effectively downregulate GRP78 expression **A.** Further studies indicated that GRP78 overexpression instigated by FD was mechanistically linked to the increased cleavage of ATF-6α (90 kDa) into active subunit ATF-6α (50 kDa) and nuclear translocation to release GRP78 from its conjugated complex **B.** Additionally, FD condition could also evoke the upregulation of Survivin in SK-Hep-1, but not in Hep-G2 cells **C.** Both FD-induced upregulation of GRP78 and Survivin could ultimately confer resistance to apoptosis by alleviating ROS production **D.**

Cultivation of SK-Hep-1 cells under FD condition could trigger the upregulation of ER stress chaperone protein 78 (GRP-78) expression. The underlying mechanism associated with this observed phenomenon was demonstrated to be mechanistically linked to the increased cleavage of ATF-6α (90 kDa) transcription factor to its active subunits (50 kDa) and nuclear translocation to release GRP-78 from its conjugated complex (Figure 2B). Along this same vein, FD condition could also capacitate SK-Hep-1 cells to upregulate Survivin expression which in turn can cooperatively act as the inhibitors for caspase 3 expression (Figure 2C). The functional attribute of overexpressed GRP78 was demonstrated to serve as a ROS sinker which alleviated the ROS production to evade apoptotic lethality as reflected by flowcytometric data using DCF-DA as the probe (Figure 2D).

### GRP78 silencing of SK-Hep-1 cells alleviates MDR

**Figure 3:**
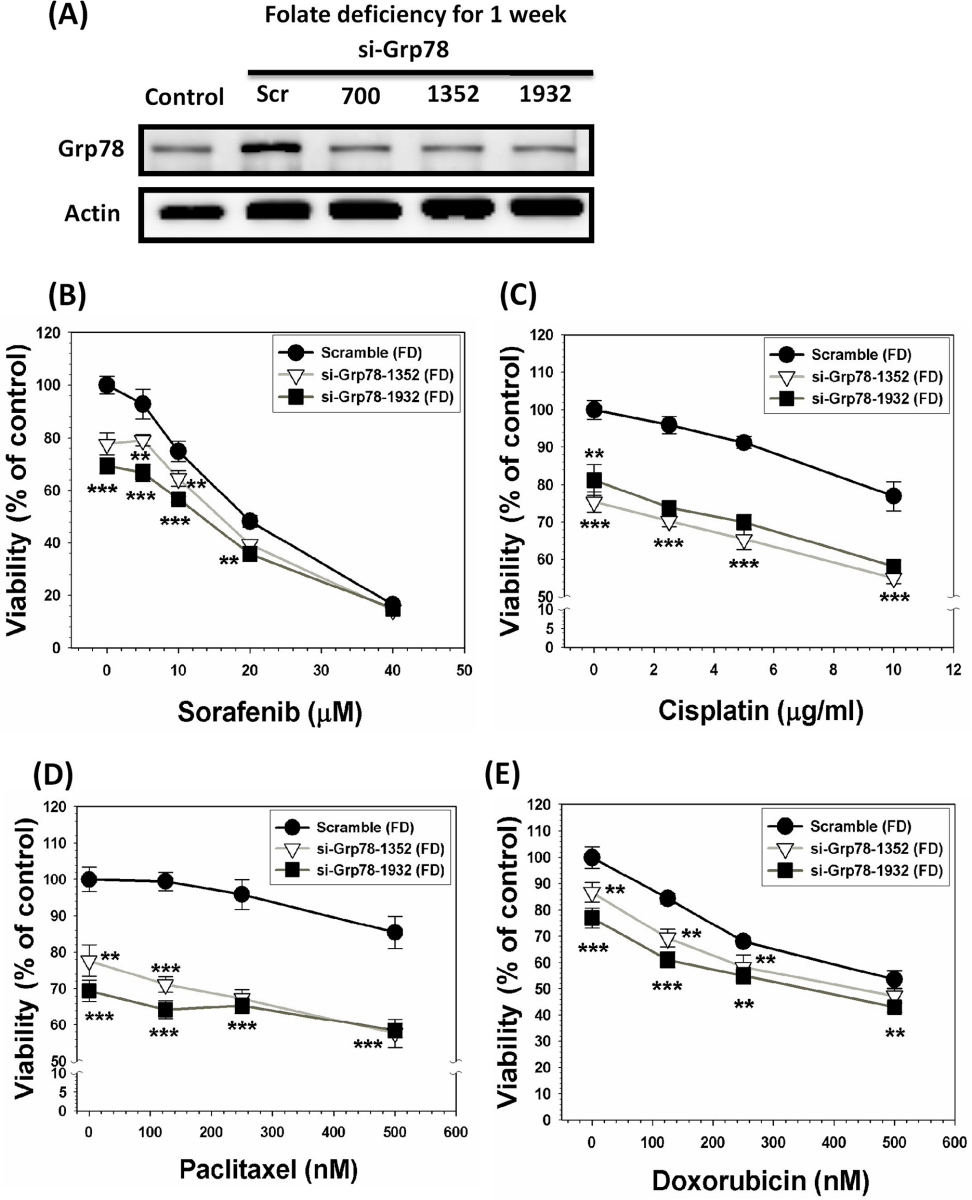
GRP78 silencing of SK-Hep-1 cells alleviates MDR acquisition attribute SK-Hep-1 cells were cultivated under FD condition for one-week to allow the induction of GRP78. Subsequently, GRP78 induced by these cells were allowed to be knockdowned by siRNA technique in varying target sites that could be vividly seen from the top of Western blotting diagrams **A.** The scramble and GRP78 knockdown (KD) cells were then treated with a group of anti-cancer drugs including sorafenib **B.** cisplatin **C.** paclitaxel **D.** and doxorubicin **E.** with varying concentrations used as indicated. Our data indicate that without exception, all the drug-treated GRP78 KD cells were substantially more vulnerable to be eradicated as compared to scramble cells. These results clearly indicate that GRP78 *per se* is a genuine chemoresistant effector. **p < 0.01; ***p < 0.001.

To further confirm that GRP78 is a genuine chemoresistant effector, SK-Hep-1 cells were grown under FD condition for one-week in order to allow GRP78 induction. Subsequently, GRP78 thus induced was allowed to be knockdowned (KD) by lentivirus-mediated knockdown technique. The scramble and two types of GRP78 KD cells were then treated with a group of anti-cancer drugs including sorafenib, cisplatin, paclitaxel and doxorubicin. Our results indicate that without exception, all the drug-treated GRP78 KD cells were substantially more susceptible to be eradicated by these drugs as compared to scramble cells (Figure 3). These data unequivocally demonstrate that GRP78 *per se* is a legitimate chemoresistant effector. **p < 0.01; ***p < 0.001.

### Direct targeting of either forcedly expressed or constitutively endowed GRP78 with EGCG effectively improves the eradicating efficiency of HCC cells

**Figure 4:**
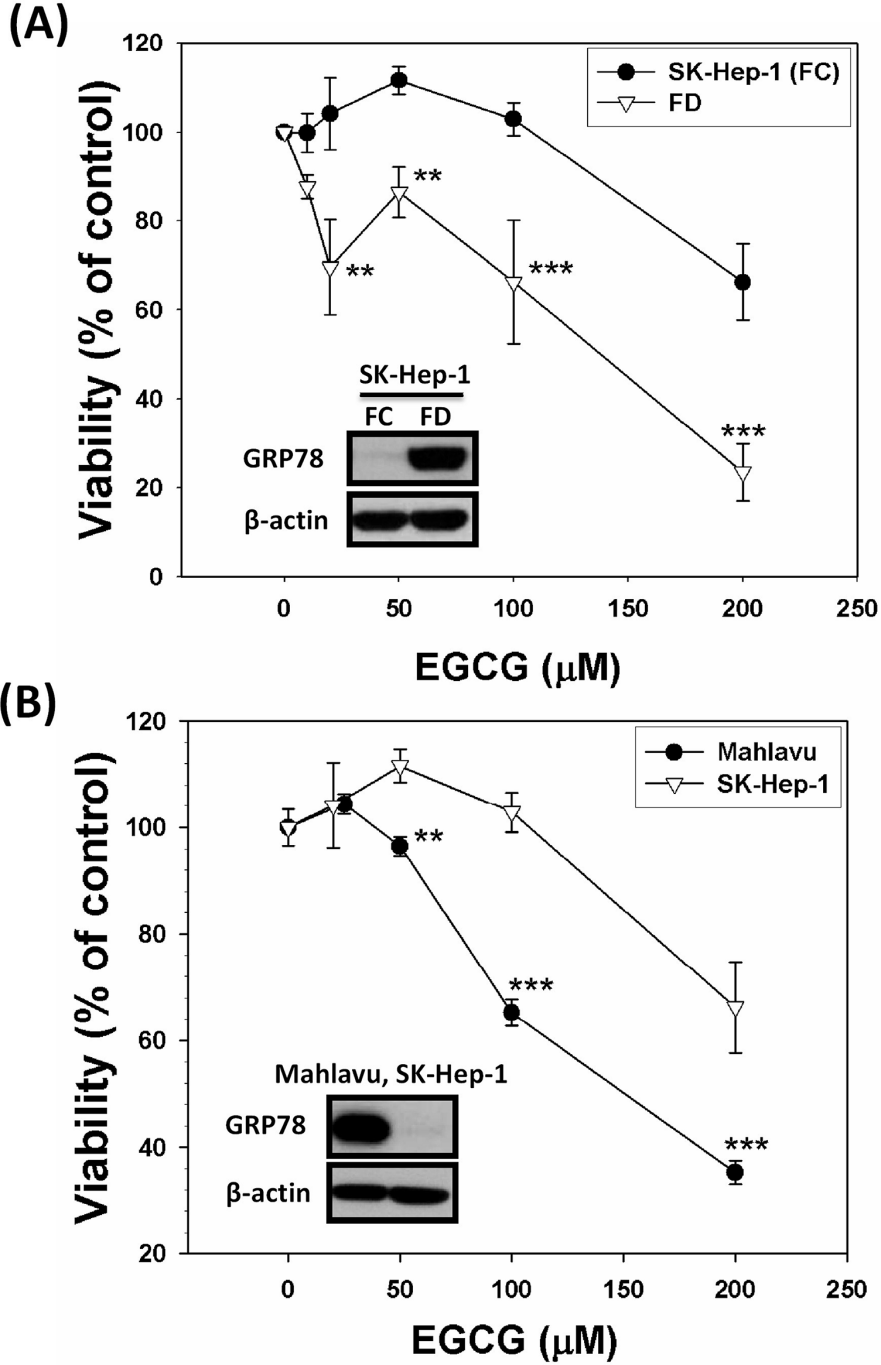
Direct targeting strategy using EGCG, an established binder for GRP78, veritably increases the eradicating efficiency of GRP78-expressing cells SK-Hep-1 cells were cultivated under FD condition for 2-week to allow overexpression of GRP78 and then treated with various concentrations of EGCG. This targeting strategy improved the eradicating efficiency of GRP78-expressing SK-Hep-1 cells by EGCG. **A.** Similarly, Mahlavu cells, a genetically endowed GRP78-expressing subline, were shown to be equally vulnerable to EGCG-instigated eradication as compared to GRP78-non expressing SK-Hep-1 counterparts **B.** **p < 0.01; ***p < 0.001.

In order to evaluate the effectiveness of direct targeting of GRP78 with its binder EGCG for the eradicating efficiency, several types of experiments were performed. First, we purposely grew SK-Hep-1 cells under FD condition to allow these cells to overexpress GRP78. Subsequently, these cells were treated with various concentrations of EGCG and continued to cultivate for an additional three days. Our data clearly demonstrated that only GRP78-overexpressing SK-Hep-1 cells (forcedly expressed subline) were particularly sensitive to be eradicated by EGCG treatment (Figure 4A). To confirm this applicability, we then tested the effect of EGCG on HCC Mahlavu cells genetically endowed with overexpressed GRP78. Again, a similar effect of EGCG could be reproduced (Figure 4B). **p < 0.01; ***p < 0.001.

### Constitutively overexpressed GRP78 efficaciously confers MDR attribute

**Figure 5:**
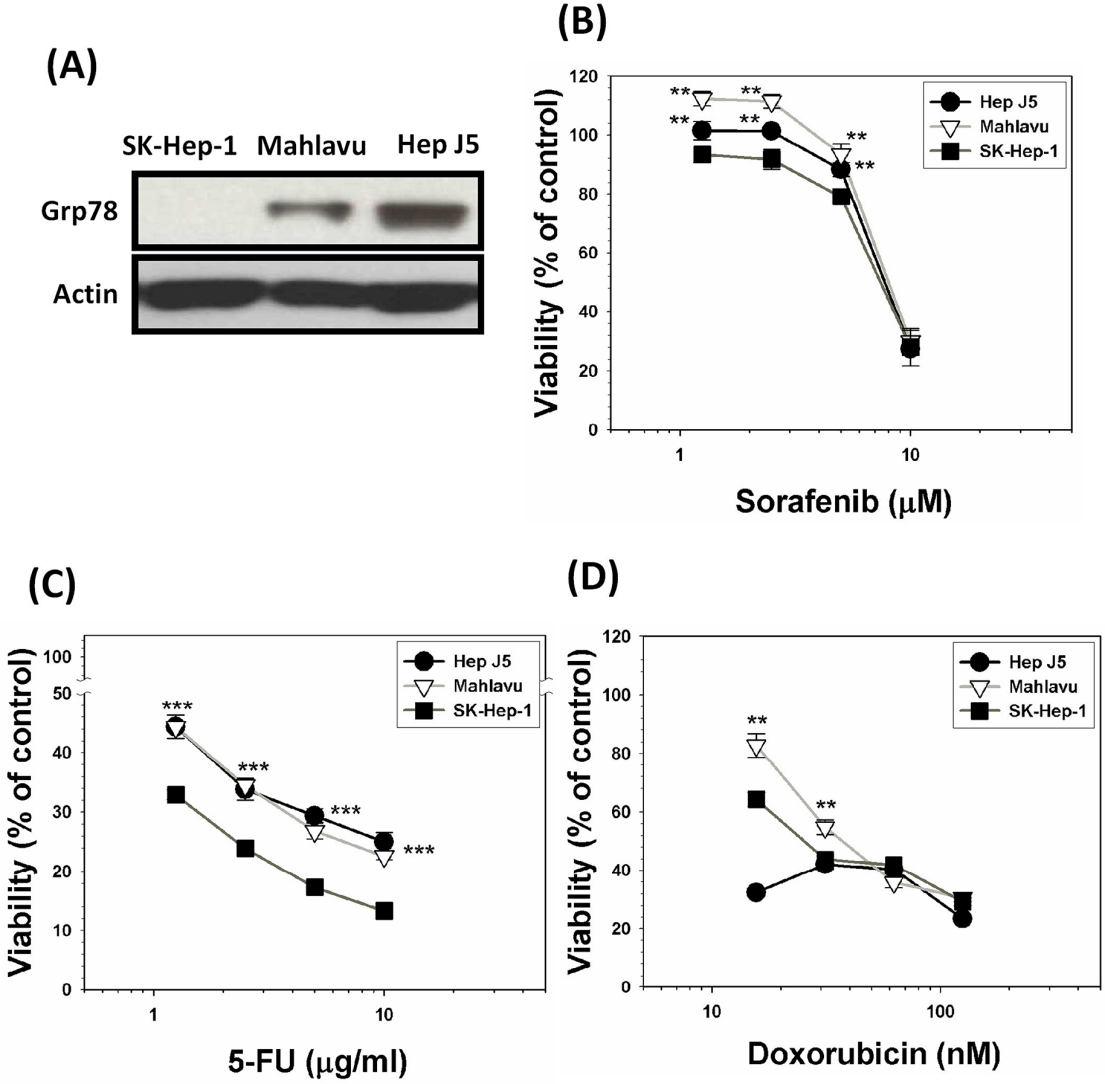
Constitutively overexpressed GRP78 confers stronger capacity of drug resistability **A.** A group of HCC subclone variants representing GRP78-overexpressing subline (Mahlavu and Hep J5) and its non-expressing counterpart (SK-Hep-1) were used to compare for their sensitivities toward a group of anti-cancer drugs including sorafenib **B.** 5-fluorouracil (5-FU) **C.** and doxorubicin **D.** by SRB method. Our data indicated that GRP78-overexpressing subclones exhibited stronger capacity of resistability against all three drugs being tested as compared to their non-expressing SK-Hep-1 cells. This finding clearly implied that constitutively overexpressed GRP78 was also a contributing factor for the MDR acquisition. **p < 0.01; ***p < 0.001.

Two HCC subclone variants representing GRP78-overexpressing (Mahlavu and Hep J5) sublines and its non-expressing counterpart (SK-Hep-1) were used to compare their sensitivities toward a group of anti-cancer drugs including sorafenib, 5-fluorouracil (5-FU), and doxorubicin. Our data indicated that GRP78-overexpressing subclones were substantially more resistant to these drugs as compared to their non-expressing SK-Hep-1 cells (Figure 5). This finding implied that constitutively overexpressed GRP78 was also a contributing factor for the MDR acquisition. **p < 0.01; ***p < 0.001.

### Either GRP78 or survivin silencing discloses two types of drug resistance mechanisms

**Figure 6:**
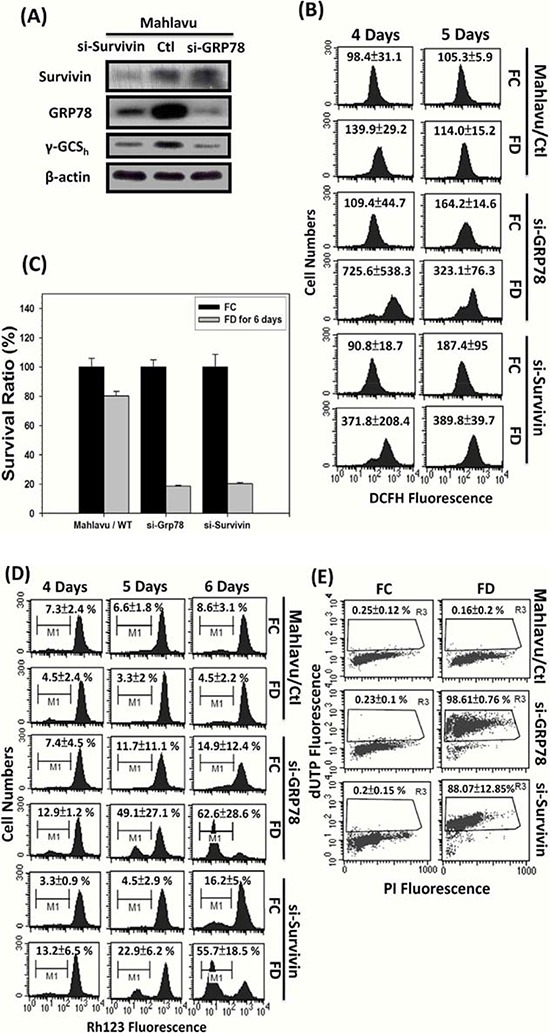
Evidence that either GRP78 or Survivin can confer Mahlavu cells the ability to evade from FD-induced apoptotic lethality In order to delineate the possible role of GRP78 and Survivin in MDR acquisition, we generated GRP78 knockdown (si-GRP78) and Survivin knockdown (si-Survivin) cells using small interfering RNA technique **A.** First, we observed that Mahlavu cells with constitutively overexpressed GRP78 and Survivin were highly tolerable to FD-induced oxidative-nitrosative stress (ONS). However, both si-GRP78 and si-Survivin cells were shown to be extremely sensitive to FD-induced ONS-mediated cell death **B** and **C.** Further flowcytometric studies revealed that either GRP78 or Survivin alone could serve as ROS sinker via alleviating ROS generation and maintaining the integrity of mitochondrial membrane potential (ΔΨm) during an episode of FD condition **D.** Consequently, with the protection of either GRP78 or Survivin, Mahlavu cells could capacitate themselves to evade from FD-induced apoptotic lethality **E.**

To delineate the role of either GRP78 or Survivin in the process of MDR acquisition, we knockdowned (KD) GRP78 or Survivin in Mahlavu cells using siRNA interfering technique. Two distinct alterations of functional attributes of the KD cells were uncovered. First, both GRP78 and Survivin silencing were associated with the downregulation of γ-GCS_h_, a catalytic subunit of intracellular GSH biosynthesis, indicating that both effectors might influence drug resistant attribute by enhancing GSH biosynthesis (Figure 6A). Second, both GRP78 and Survivin silencing drastically increased the generation of ROS during a 4-day cultivation period under FD condition implying that both effectors functioned as a ROS sinker (Figure 6B). Furthermore, both GRP78 and Survivin KD cells severely reduced their abilities to survive under FD condition (Figure 6C). Consequently, both GRP78 and Survivin KD cells were found to undergo depolarization of mitochondrial membrane potential (ΔΨm) (Figure 6D) leading to apoptotic lethality as reflected by the drastic increase of TUNEL-positive cells from 0.16 ± 0.2 to 98.6 ± 0.8% (for GRP78 KD cells) and 88.07 ± 12.85% (for Survivin KD cells), respectively (Figure 6E).

### Silencing of genetically endowed GRP78 or survivin significantly mitigates MDR acquisition attribute of Mahlavu cells

**Figure 7:**
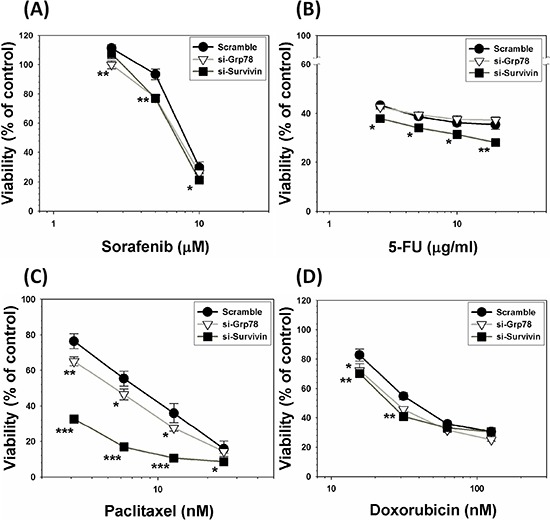
Silencing of genetically endowed GRP78 or Survivin alleviates MDR acquisition attribute of Mahlavu cells Both GRP78 and Survivin of HCC Mahlavu cells were subjected to be knockdowned by siRNA technique. Subsequently, scramble, GRP78 KD and Survivin KD cells were treated with a group of anti-cancer drugs including sorafenib **A.** 5-FU **B.** paclitaxel **C.** and doxorubicin **D.** Our data indicate that without exception, all the drug-treated GRP78 KD or Survivin KD cells exhibited increased vulnerability to be eradicated by these drugs as compared to scramble cells indicating that both GRP78 and Survivin are genuine chemoresistant effectors. *p < 0.05; **p < 0.01; ***p < 0.001.

To verify if either constitutively overexpressed GRP78 or Survivin is a genuine chemoresistant effector, we used siRNA interfering technique to knockdown both effectors of Mahlavu cells. Subsequently, scramble, GRP78 KD and Survivin KD cells were then treated with a group of anti-cancer drugs including sorafenib, 5-FU, paclitaxel and doxorubicin. Our data unequivocally demonstrated that without exception, all the drug-treated GRP78 KD or Survivin KD cells were substantially more vulnerable to be eradicated by these drugs as compared to scramble cells (Figure 7). This finding further demonstrates that both GRP78 and Survivin are a pair of genuine chemoresistant effectors.

## DISCUSSION

Cellular redox homeostasis is maintained by the balance between ROS generation and elimination. Exogenous agents that increase ROS generation or decrease antioxidant capacity shift the redox balance in favor of an overall increase in the levels of ROS, which can ultimately induce cell death [25, 26]. This scenario was observed in hepatoma Hep G2 cells, a well-differentiated and least oxidatively stressed subclone variant, which underwent apoptosis when exposed to folate deprivation (FD)-induced ONS, as reported previously by our group [18, 27, 28]. A similar scenario has been reported [21, 22]. However, in sharp contrast to Hep G2 cells, three HCC cell lines including SK-Hep-1, Mahlavu and Hep J5 were found to be capable of evading FD-induced apoptosis and becoming MDR against a group of ROS-producing anti-cancer agents including doxorubicin, paclitaxel, cisplatin and sorafenib (Figure 1). Interestingly, this transient drug resistance can be overcome by supplementation of folate to the culture medium. This finding has important clinical application.

Next, we investigated mechanism(s) of survival under FD-induced ONS. Recent work showed that ROS can cause redox adaptation, survival under ROS stress and resistance to certain anti-cancer agents [26]. As shown by Landriscina et al. [25], one of mechanism is heat-shock protein (HSP) expression. Elevated HSP expression prevents spontaneous apoptosis and apoptosis caused by therapeutic agents. We hypothesized that FD-induced ONS activates HSP pathways that confers MDR. Indeed, we found that SK-Hep-1 cells, but not Hep G2 cells, unilaterally activate chaperone protein 78 (GRP78). GRP78 plays role in the ER-Ca^2+^ balance via transmembrane ER stress sensor and controls protein folding [29–31]. As a biomarker of unfolded protein response (UPR), GRP78 overexpression has been associated with chemoresistance in a variety of cancers including head and neck squamous carcinoma, glioma, melanoma, breast and hepatoma [32–36]. Resupplementation of folate can effectively downregulate GRP78 expression in SK-Hep-1 cells, indicating that folate can be used to overcome drug resistance by FD. This finding underscores the clinical significance of monitoring the folate status prior to the therapeutic intervention. Our data demonstrated that FD-induced ONS probably upregulates an ER stress-associated GRP78 pathway via an enhanced cleavage of ATF6α (90 kDa) transcription factor to its active ATF6α (50 kDa) subunit and nuclear translocation to release GRP78 from its conjugated complex (Figure 2). FD was also induced overexpression of Survivin, which could enable SK-Hep-1 cells to evade apoptosis by inhibiting caspase 3 activation (Figure 2C). Both GRP78 and Survivin could then cooperatively alleviate ROS production [24] (Figures 2D and 6B).

Our work can help to improve tumor response and patient survival. We hypothesized that agents capable of abrogating such adaptation mechanisms in combination with conventional chemotherapy should improve therapeutic outcomes. We first utilize (−)-Epigallocatechin gallate (EGCG), one of the major constituents of green tea [37–41]. GRP78 overexpressed SK-Hep-1 cells after cultivating under FD condition for 2-week could be re-sensitized by EGCG to be eradicated more efficaciously than control cells without GRP78 expression. To further confirm this notion, we utilized Mahlavu cell, which is genetically endowed with overexpressed GRP78. When both Mahlavu and SK-Hep-1, cultivated under folate sufficient (FC) condition, only GRP78-expressing Mahlavu cells were more susceptible to be eradicated by EGCG targeting strategy. Besides GRP78-targeting strategy, we also performed the knockdown study via small RNA interfering technique on forced GRP78 overexpression induced by growing SK-Hep-1 cells under FD condition. As indicated in Figure 3, GRP78 KD cells (si-GRP78–1352 and si-GRP78–1932) were extremely vulnerable to be eradicated by all four anti-cancer drugs tested when compared to the scramble cells. Both GRP78 targeting strategy and Grp78 knockdown studies demonstrate that Grp78 is a contributing factor for MDR.

To test the universality of this finding, we thus set out to evaluate the relative sensitivity of two types of GRP78-overexpressing subclones (Mahlavu and Hep J5) and SK-Hep-1 (Grp78-nonexpressing subclone) cells toward a group of ROS-producing anti-cancer drugs including doxorubicin, 5-fluorouracil and sorafenib. Our data showed that indeed GRP78 plays a pivotal role in MDR acquisition because SK-Hep-1 cells were relatively more vulnerable to be eradicated by these ROS-producing anti-cancer drugs owing to the deficiency of GRP78 expression (Figure 5). In summary, GRP78, either constitutively endowed or forcedly expressed, could indeed contribute to the survival by defending exogenously ROS insults.

To further obtain evidence to substantiate the mechanistic link between GRP78, Survivin and MDR, we performed GRP78 and Survivin silencing experiment using siRNA technique. There are two important findings. First, either GRP78 or Survivin silencing result in downregulation of heavy subunit of γ-glutamylcysteine synthetase (γ-GCS_h_), a critical catalytic enzyme responsible for the biosynthesis of GSH. Second, either GRP78 or Survivin silencing could drastically promote the production of ROS under FD condition as reflected by an approximately 3- to 6-fold increases in DCF fluorescence intensity observed flowcytometrically (Figure 6B). This finding implicates that both GRP78 and/or Survivin may play pivotal role as a ROS sinker. Consequently, elevated ROS production in cells without GRP78 or Survivin expression will ultimately provoke the depolarization of mitochondrial membrane potential (ΔΨm) leading to apoptotic lethality (Figure 6E). Along the same vein, either GRP78 or Survivin silencing could sensitize Mahlavu cells to be effectively eradicated by a group of ROS-producing anti-cancer drugs including sorafenib, 5-FU, paclitaxel and doxorubicin (Figure 7).

**Figure 8:**
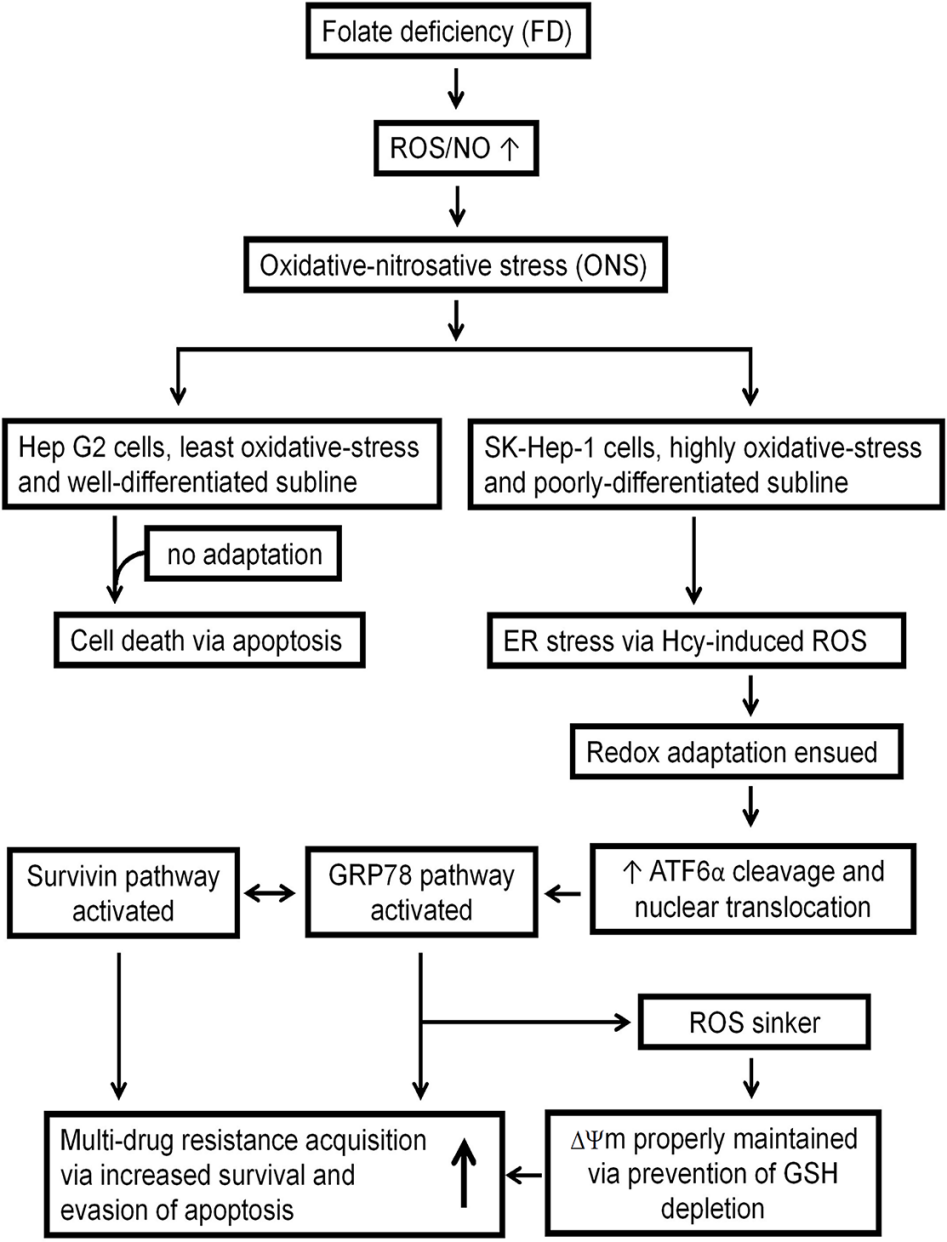
Diagrammatic representation illustrating the involvement of various pathways activated by FD-mediated ONS that conferring MDR attribute

Several conclusions can be drawn from the current studies. First, our study adds to the literature on the direct and concrete evidence linking the relationship between FD-induced ONS and enhanced MDR acquisition. Second, our findings link FD-induced ROS to redox adaptation-mediated upregulation of GRP78 and Survivin. Third, disruption of GRP78 expression with EGCG and siRNA knockdown sensitized GRP78-expressing HCC cells to be eradicated. Fourth, our results suggest that prior to chemotherapeutic treatment, monitoring of nutritional status of folate probably should be necessary. In another words, treatment with folate to prevent its deficiency may be of critically importance to achieve satisfactory result of chemotherapy. Finally, the overall interactions of various pathways activated by FD-mediated ONS, which confer MDR, can be summarized diagrammatically in Figure 8.

## MATERIALS AND METHODS

### Materials

Folate, amino acids, nucleosides, nucleotides and other chemical compounds were purchased from Sigma-Aldrich (St. Louis, MO, USA). Minimal essential medium/alpha modified (αMEM) without ribosides, ribotides, deoxyriboside, deoxyribotides, glycine, serine and folate was specially ordered and formulated by Invitogen and JRH (Lenexa, KS, USA). Fetal bovine serum (FBS) was from Biological Industries (Kibbutz, Israel). Penicillin, streptomycin, fungizone, trypsin and trypan blue were from GIBCO Laboratories (Grand Island, NY, USA).

Antibodies specific for heavy subunit of γ-glutamylcysteine synthetase (γ-GCS_h_), COX-2, Bcl-2, GRP78, ATF4, ATF6α, SP-1 (Santa Cruz Biotechnology, california, USA), Survivin, pro-caspase 3 (Cell Signaling Technology, Danvers, MA, USA), PARP-1, β-catenin (Epitomics, california, USA), and β-actin (Sigma-Aldrich, St. Louis, MO, USA) were obtained.

### Cell line and cell culture

Human hepatocellular carcinoma (HCC) cells were obtained from the National Development Center for Biotechnology (Taipei, Taiwan). A panel of 4 HCC cells, including Hep G2, SK-Hep-1, Hep J5 and Mahlavu, with distinct disparity in differentiation status were used as the experimental cell models. Cells was grown in minimal essential medium/alpha modified (αMEM) with 10% heat-inactivated FBS and cultured at 37°C in a humidified atmosphere with 5% CO_2_. αMEM was complete medium contained with folate (2 μmole/L), thymidine (36 μmole/L), hypoxanthine (36 μmole/L), glycine (600 μmole/L), serine (250 μmole/L). Pencillin (20,000 units/L), streptomycin (20 mg/L) and fungizone (2.5 mg/L) were also added to media for the elimination of contamination. To formulate folate-deficient media, folate as well as thymidine, hypoxanthine and glycine were omitted from complete media to stress substrate availability in one-carbon metabolism. To minimize exogenous folate sources, fetal bovine serum was replaced with dialyzed fetal bovine serum (dFBS), which had been dialyzed at 4°C for 16-h against 6 × 10 volumes of sterile PBS. Control medium was complete medium with 10% FBS. Therefore, HCC cells culture in folate-depleted medium (in the absence of folate and thymidine, hypoxanthine, glycine and serine) are designated as folate-deficient cells (FD). HCC cells culture in the control medium are referred to as folate control cells (FC).

### Preparation of total protein and nuclear extracts

1 × 10^6^ of HCC cells were seeded in 10-cm dishes and maintained at 37°C with 5% CO_2_ for 48 h. Whole-cell lysates and nuclear extracts were collected from cells at 80% confluence. Adherent cells were scraped into 3 ml of cold phosphate-buffered saline (PBS), pelleted by centrifugation, and resuspended in 100 μl of total lysis buffer (Enzo, Lausen, Switzerland) with protease inhibitors and phosphatase inhibitors (Sigma-Aldrich, St. Louis, MO, USA). Cells were broken up by strong vortexing and incubated on ice for 10 min; these steps were repeated three times. Then, cell lysates were centrifuged at 13,000 rpm for 30 min at 4°C. The supernatants were whole-cell protein extracts.

The nuclear isolation procedure was based on the manufacturer's instructions from the Nuclei EZ Prep Kit (Sigma-Aldrich, St. Louis, MO, USA). Adherent cells were scraped into 150 μl of Nuclei EZ Lysis buffer, swollen by mild vortexing on ice for 5 min, and then centrifuged at 1000 *g* for 10 min at 4°C. Supernatants containing cytoplasmic extracts were stored at −80°C. The nuclear pellet was resuspended in 100 μl of Nuclei EZ Lysis buffer with mild vortexing on ice for 5 min to wash the pellet and then centrifugation at 1000 *g* for 10 min at 4°C. After removing the supernatant, the nuclear pellet was lysed with Nuclei EZ Storage buffer and triturated 5∼10 times with a micropipette to help break up clumps of nuclei. The final nuclear extracts in storage buffer were stored at −80°C or used for Western blot analysis.

### Western blot analysis

Protein expression was analyzed with a specific antibody. Equal amounts of protein extract (40 μg, nuclear or total extract) were boiled for 6 min in sample buffer [300 mM Tris-HCl (pH 6.8), 3.85% DTT, 9% sodium dodecylsulfate (SDS), 25% glycerol, and 0.033% bromophenol blue], subjected to 8%∼12% SDS-polyacrylamide gel electrophoresis (PAGE), and transferred to polyvinylidene difluoride (PVDF) membranes (Millipore, Billerica, MA, USA). Membranes were probed with first antibodies against γ-GCS_h_ (1: 500), actin (1: 10^4^), COX-2 (1: 500), Bcl-2 (1: 250), GRP78 (1: 1000), ATF4 (1: 250), ATF6α (1: 250), Survivin (1: 1000), pro-caspase 3 (1: 1000), PARP-1 (1: 1000), β-catenin (1: 1000) and SP-1 (1: 500). After washing in TBST buffer, the membrane was further incubated with anti-rabbit secondary antibody (Chemicon, Millipore, Billerica, MA, USA) for 1-h. Detection was performed by enhanced chemiluminescence (ECL; Millipore, Billerica, MA, USA) after incubation with a horseradish peroxidase (HRP)-conjugated secondary antibody. β-actin was used as the loading control.

### Cell viability assay

Cells were seeded in a 6-well plastic plate and exposed to 1, 5 μM doxorubicin (Sigma-Aldrich, St. Louis, MO, USA), 20, 40 μg/ml cisplatin (Platinol^®^), 1, 5 μg/ml paclitaxel (Taxol^®^), 20, 40 μM sorafenib (*Nexavar*^®^) in duplicate for 48-h in at least two independent experiments (*n* = 4). Cell viability was assayed by the sulforhodamine B (SRB) method. Growth inhibition was calculated as the percentage of surviving cells in drug-treated versus untreated cells (which were incubated with FD for 1 or 2 weeks). In SRB method, cultures fixed with trichloroacetic acid and were stained for 30 minutes with 0.4% (w/v) SRB (Sigma-Aldrich, St. Louis, MO, USA) dissolved in 1% acetic acid. Unbound dye was removed by two washes with 1% acetic acid, and protein-bound dye was extracted with 10 mM unbuffered Tris base [tris(hydroxymethyl) aminomethane] for determination of optical density in 515 or 540 nm by ELISA reader.

### Lentivirus-mediated knockdown of GRP78 expression

Small hairpin RNA (shRNA) targeting HSPA5 (GRP78) (si-GRP78–700: GCCATGGTTCTCACTAAAA, sh-GRP78–1352: GCTCGACTCGAATTCCAAA and si-GRP78–1932: GCGCATTGATACTAGAAAT) were cloned into the pLV-H1-EF1a-GFP-Puro vector and packaged in a lentivirus (Biosettia, USA). Lentivirus-containing medium and polybrene were added to cultures of folate deficiency-treated SK-Hep-1 cells with puromycin selection.

### Generation of GRP78 or survivin knockdown mahlavu cells

The GRP78 and Survivin knockdown of Mahlavu cells were carried out according to the procedures of Chang et al. [23, 24]. Briefly, the target sequence for the human GRP78 mRNA was 5′-AAGGTTACCCATGCAGTTGTT-3′ and the human Survivin mRNA was 5′-TGGGAGCCA GATGACGACC-3′. The scrambled siRNA sequence was 5′-AAGGTGGTTGTTTTGTTCACT-3′. The GRP78 siRNA, Survivin siRNA and scrambled siRNA were inserted into the pSUPERIOR vector and transfected into the cells. We applied 1 pulse for 20 milliseconds under a fixed voltage of 1.4 kV on a pipette-type microporator Neon (Invitrogen Life Technologies). Cells that were successfully transfected were selected by antibiotic resistance.

### Flowcytometric measurement of intracellular ROS and mitochondrial membrane potential (ΔΨm)

All fluorescence probes were purchased from Sigma-Aldrich (St. Louis, MO) unless otherwise specified. Intracellular ROS production was measured using DCFH-DA probe. Briefly after cells were cultivated under FD condition, the culture medium was then replaced with new FD medium and followed by the incubation with 10 μM DCFH-DA for 30 min in the dark. Cells were then washed once with PBS, detached by trypsinization and collected by centrifugation and re-suspended in PBS.

To measure cellular *mitochondrial* membrane *potential* (ΔΨm), FC or FD cells were incubated with 5 μM rhodamine 123 for 30 min in the dark. Cells were then washed twice with PBS, detached by trypsinization and collected by centrifugation and re-suspended in PBS. The fluorescence intensity was measured using a FACS-Calibur flowcytometer (BD Biosciences, San Jose, CA) and analyzed using CellQuest software.

### TUNEL assay

Apoptotic cell death was assayed by using an Apo-BrdU *in situ* DNA fragmentation assay kit (Promega, Medison, WI, USA). This kit measures the fragmented DNA of apoptotic cells by catalytically incorporating fluorescein-12-dUTP at 3′-OH DNA ends using the terminal deoxynucleotidyl transferase enzyme. The fluorescein-12-dUTP-labeled DNA can then be quantitiated by flowcytometry. In this study, Mahlavu cells were cultivated in either FC or FD condition for six days and the fluoressein-12-dUTP-labeled fragmented DNA of apoptotic cells were quantified using Becton-Dickinson FACS-Calibur flow- cytometer.

### Statistical analysis

Data were expressed as the mean ± standard deviation (SD) from at least two independent experiments. A paired Student's *t*-test was used to determine the differences between control and treatment groups (Sigma plot 8.0 software). Differences at a level of *p* < 0.05 for Student's *t*-test were considered statistically significant.

## SUPPLEMENTARY FIGURE


