# Supplementary material for: Folate deficiency-triggered redox pathways confer drug resistance in hepatocellular carcinoma
Source: Oncotarget. 2015 Jun 27;6(28):26104–18. doi: 10.18632/oncotarget.4422 (PMC4694889; doi:10.18632/oncotarget.4422)
Supplement: Supplementary file 1 [file oncotarget-06-26104-s001.pdf]

SUPPLEMENTARY FIGURE

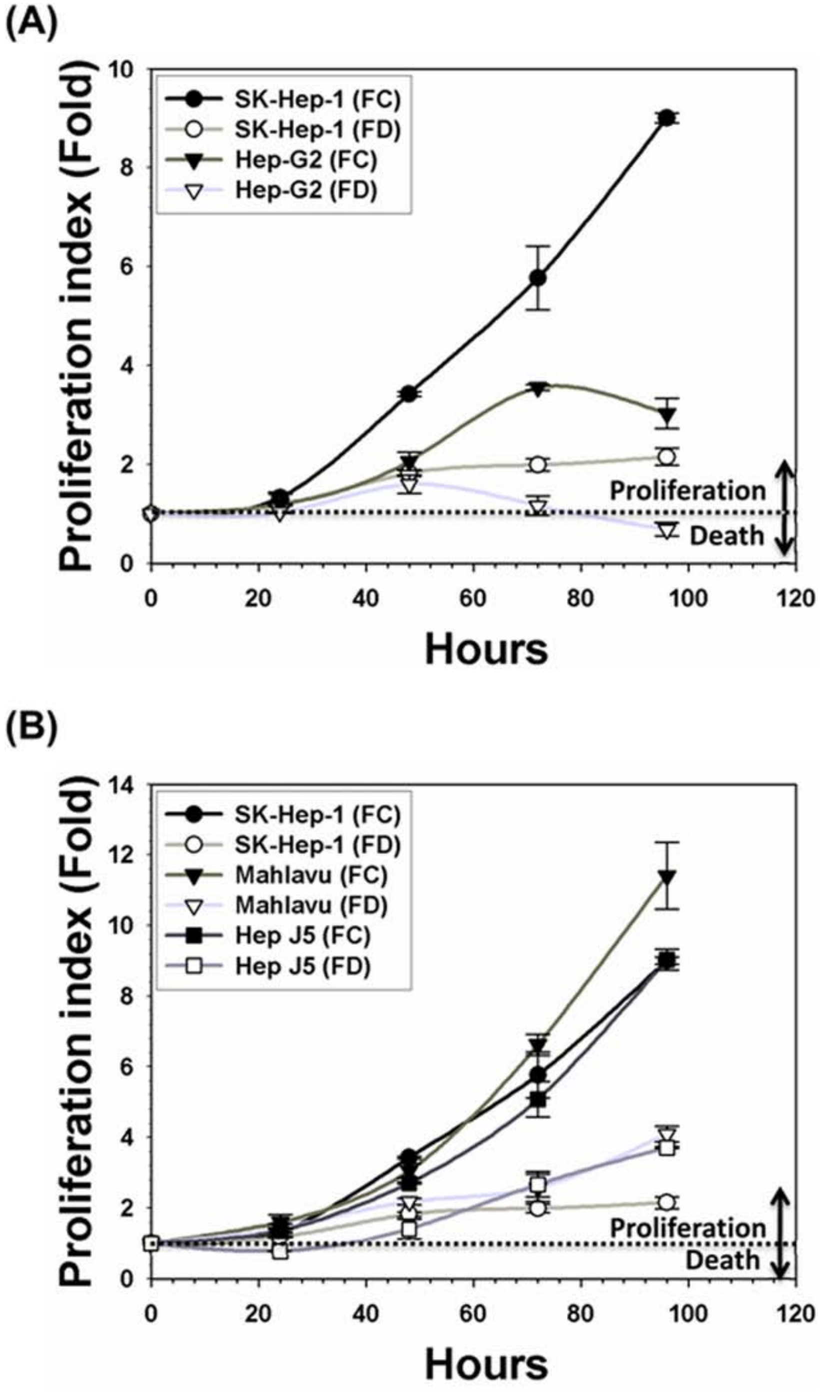

Supplementary Figure S1: The proliferative ability of a group of HCC cells cultivated in the presence or absent of folate compared. Hep G2 (5×10^5/well) and SK-Hep-1 (2×10^5/well) were cultivated in 6-well plates for 24-, 48-, 72- and 96-h and the cell numbers of each plate were then counted by the trypan blue method. The proliferation index was expressed as fold of the seeding cell number. The dotted line represented one-fold. In comparison, the viability of Hep G2 cultivated under FD condition at 72-h was less than one fold of seeding cell number **A.** Conversely, SK-Hep-1 cells cultivated under FD condition for 72-h and continued to grow albeit in a slower pace. In a similar fashion, both Mahlavu and Hep J5 cells (2×10^5/well) in FD condition were shown to be equally resistant to FD-induced cell demise and continued to grow slowly **B.** Collectively, our data implicate that three out of the four HCC subclone variants including SK-Hep-1, Mahlavu and J5 cells which can be classified as redox adaptation-prone phenotype capable of surviving under FD condition. In contrast, Hep G2 cell is a redox adaptation-null phenotype which will undergo apoptosis under FD condition due to its inability of inducing prosurvival pathways.
